# Developing and validating a clinically actionable prediction tool for Parkinson disease using explainable machine learning with multidimensional national data: A cross-sectional study

**DOI:** 10.1097/MD.0000000000047983

**Published:** 2026-03-20

**Authors:** Li Ke, Ying Li, Sili Jiang, Lei Zhao

**Affiliations:** a Department of Cerebrovascular Diseases, Suining Central Hospital, Suining, Sichuan Province, China.

**Keywords:** boruta algorithm, least absolute shrinkage and selection operator (LASSO), machine learning (ML), minimum redundancy maximum relevance, mRMR, Parkinson disease (PD)

## Abstract

Parkinson disease (PD) is a rapidly growing neurodegenerative disorder that presents a significant public health challenge in aging societies, particularly in China. Although genetic, clinical, and environmental risk factors have been well-established, the predictive value of multidimensional factors (encompassing socioeconomic determinants, prodromal functional impairments, and lifestyle) remains underexplored in Chinese populations. We used data from the China Health and Retirement Longitudinal Study (n = 13,649; PD cases = 269) to develop and validate a machine learning prediction model for PD. Candidate predictors covered cognition, socioeconomic status, comorbidities, and lifestyle. Feature selection was performed using an ensemble approach (boruta, least absolute shrinkage and selection operator, and minimum redundancy maximum relevance), followed by extreme gradient boosting (XGBoost) model development with Bayesian hyperparameter optimization. Data were randomly split into a training set (70%) and a validation set (30%) for internal validation. Model performance was evaluated using area under the receiver operating characteristic curve, decision curve analysis, sensitivity, specificity, positive predictive value, negative predictive value, and F1-score. Model interpretability was evaluated using SHapley Additive exPlanations. Key predictors included cognitive impairment (memory and executive deficits), physical inactivity (low metabolic-equivalent scores), socioeconomic indicators (retirement status and financial support), educational attainment, and comorbidities (e.g., liver disease and wrist pain). In the validation set, the optimized XGBoost model achieved an area under the curve of 0.967 (95% confidence interval 0.962–0.972), sensitivity 82.0%, specificity 94.7%, positive predictive value 81.5%, negative predictive value 94.9%, and F1-score 81.7. SHapley Additive exPlanations analyses supported the consistency and relative importance of these predictors. An explainable XGBoost model integrating multidimensional national data showed excellent internal validation performance for identifying individuals with PD in China Health and Retirement Longitudinal Study. This tool may help support PD risk stratification in Chinese populations; external validation and prospective evaluation are warranted before clinical implementation.

## 1. Introduction

Parkinson disease (PD) is recognized as the second most prevalent neurodegenerative disorder associated with aging.^[1,2]^ It demonstrates an age-dependent escalation in both incidence and prevalence and is pathologically characterized by the loss of dopaminergic neurons in the substantia nigra and the formation of Lewy bodies.^[2,3]^ According to the 2021 Global Burden of Disease, Injury, and Risk Factors study, it is estimated that over 11.8 million individuals worldwide are affected by PD.^[4]^ Notably, China had the highest age-standardized incidence and prevalence rates of PD among G20 countries (excluding the European Union).^[5]^ By the year 2030, it is projected that 50% of individuals globally diagnosed with PD will be from China.^[6]^ This condition manifests a variety of motor symptoms, including tremors, rigidity, reduced mobility, and balance impairments, alongside nonmotor symptoms such as s loss or decrease of sense of smell, cognitive decline, depression, sleep disturbances, and discomfort.^[1,3]^ These multisystemic features exert a substantial effect on individuals and society as a whole.^[7,8]^ Despite advancements in symptomatic therapies, PD remains incurable.^[9]^ Consequently, early identification of at-risk populations is essential to optimize clinical management and delay disease progression.

Recent research has increasingly focused on identifying multidimensional risk characteristics that integrate genetic, environmental, and clinical predictive factors. Genome-wide association studies have identified 90 risk loci associated with the pathogenesis of PD, particularly in pathways involving synaptic transmission and lysosomal function.^[10]^ Extensive research has been conducted on potential biomarkers associated with PD, including neuroimaging, cerebrospinal fluid analysis and serum biomarkers.^[11,12]^ From an epidemiological perspective, the association between PD and complications such as diabetes, cardiovascular disease, and depression suggests a shared pathophysiological mechanism.^[13–15]^ However, these models often overlook socioeconomic determinants and functional impairments, which may manifest several years prior to clinical diagnosis. Despite regional differences in the incidence of PD,^[16,17]^ there remains a paucity of studies examining risk factors specific to China.

Current evidence indicates that machine learning (ML) can effectively analyze complex datasets, including clinical data, genetic information, and imaging features, thereby facilitating early detection and diagnosis of diseases.^[18]^ Our study leveraged nationally representative longitudinal data from the China Health and Retirement Longitudinal Study (CHARLS) to capture multidimensional socioeconomic, health, and functional variables. We aimed to apply ML algorithms to elucidate the relationships between these variables and PD.

## 2. Methods

### 2.1. Study design and data source

**Figure 1.**
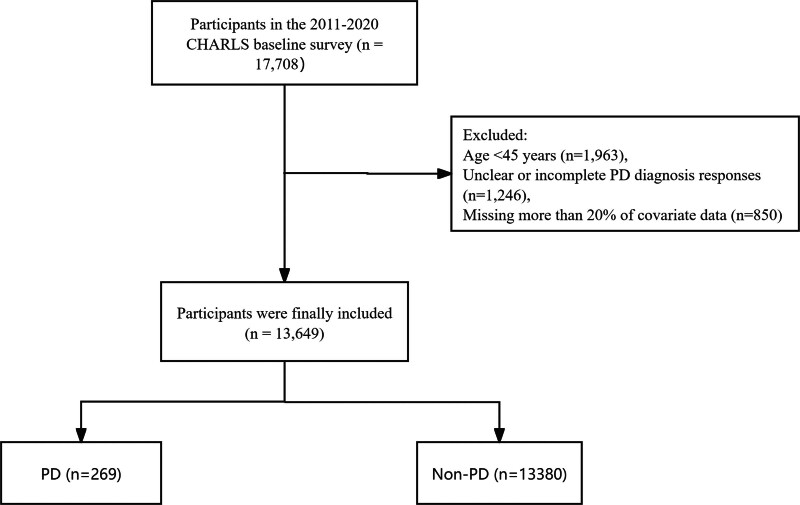
Study flow chart.

This study utilized data from the CHARLS, a nationally representative longitudinal survey conducted to understand health dynamics, socioeconomic conditions, and family structures among Chinese residents aged 45 years and older. CHARLS employs multistage stratified probability sampling to collect extensive demographic, health-related, social, and economic data. Data collection involved structured questionnaires and clinical assessments performed by trained personnel. All participants in the CHARLS study provided written informed consent, the consent process was approved by the Peking University Institutional Review Board (IRB00001052-11015). As this study uses de-identified public data from CHARLS, additional consent was not required per China’s ethical regulations for secondary data analysis. A total of 17,708 individuals took part in the 2011 to 2020 CHARLS baseline survey. After excluding participants younger than 45 years (n = 1963), respondents with missing or ambiguous answers to the physician-diagnosed PD question (n = 1246), and those with >20% missing values in mandatory covariates (n = 850), 13,649 participants (76.9 % of the baseline sample) were included in the analysis. The dataset comprised 13,649 participants, including 269 PD cases and 13,380 non-PD controls. Figure 1 illustrates the data integration process.

Data were randomly partitioned into 2 sets for robust model development and validation: a training set (n = 9650, approximately 70%) used for model building and parameter tuning, and a validation set (n = 3999, approximately 30%) for evaluating the model’s predictive performance.

### 2.2. Data preprocessing

The preprocessing stage was critical to ensure data quality and reliability. Initial preprocessing included rigorous completeness checks, identifying and excluding variables with substantial missing data (over 20%). For variables with lower levels of missing data, missing values were handled using median substitution for continuous variables or mode substitution for categorical variables, based on the data type and distribution.

Continuous variables were normalized to standardize the data and reduce biases associated with different scales. Normalization involved scaling data to have a mean of zero and a standard deviation of 1, which aids in model convergence and interpretation.

### 2.3. Variable selection methods

#### 2.3.1. Boruta algorithm

The Boruta algorithm was employed for feature selection due to its capacity to handle large and complex datasets effectively. Boruta operates by running random forest classifiers iteratively, creating additional artificial features termed “shadow features” that mimic original features by shuffling values. The importance of each original feature was measured against these shadow features. Variables consistently performing better than shadow features were categorized as “important,” whereas those with inconclusive performances after multiple iterations were labeled “tentative.” The iterative process was conducted until clear delineations were obtained or a set threshold of iterations was reached.

#### 2.3.2. Least absolute shrinkage and selection operator (LASSO)

LASSO regression was utilized for its dual function of regularization and feature selection, particularly effective in high-dimensional datasets. LASSO applies a penalty equal to the absolute value of regression coefficients, shrinking some coefficients to zero and thereby effectively selecting variables most strongly associated with the outcome. Cross-validation methods were employed to identify the optimal lambda (λ) value, defined as lambda.min, which minimizes prediction error. Variables retained at lambda.min represented those with significant predictive power.

#### 2.3.3. Minimum redundancy maximum relevance (mRMR)

The mRMR algorithm was incorporated into the feature selection workflow to ensure maximal relevance of variables to Parkinson diagnosis and minimal redundancy among selected variables. This 2-step evaluation was critical to improving predictive accuracy and computational efficiency by eliminating redundant predictors.

### 2.4. Model development and evaluation

We constructed an XGBoost (extreme gradient boosting) model for PD prediction, utilizing the selected features from the feature selection process. XGBoost was chosen for its strong predictive performance, scalability, and ability to handle large datasets. Hyperparameters were optimized through grid search and cross-validation to ensure the best possible model configuration for prediction accuracy. Model performance was evaluated using the receiver operating characteristic (ROC) curve and area under the curve (AUC). The ROC curve allows for an assessment of the trade-off between sensitivity and specificity, while the AUC provides a single value indicating the model’s overall discriminatory power. Additional metrics including accuracy, sensitivity, specificity, precision, and F1-score were also calculated to offer a comprehensive view of the model’s effectiveness. In addition, given the pronounced class imbalance between PD and non-PD participants (269 vs 13,380), we implemented strategies to reduce bias toward the majority class. Specifically, we applied the Synthetic Minority Oversampling Technique to the training set and tuned the XGBoost classifier’s class-weighting (e.g., scale_pos_weight) to improve minority-class learning. Model performance was primarily assessed using ROC–AUC and related discrimination metrics (including sensitivity and specificity), and we further reported balanced accuracy to provide an imbalance-robust summary. Beyond discrimination, we performed decision curve analysis to evaluate potential clinical utility by quantifying net benefit across a range of threshold probabilities.

The model’s generalizability was validated using the validation set (30% of the data). We also performed a 5-fold cross-validation on the training set to tune model parameters and mitigate overfitting. In K-fold cross-validation, the data was split into K subsets, and the model was trained and tested on each fold, ensuring robust validation and an average performance estimate over multiple iterations.

### 2.5. Statistical analysis

Statistical analyses were performed using R (version 4.2.1; R Foundation for Statistical Computing, Vienna, Austria). Chi-square tests were used for categorical variable analysis, and logistic regression analysis were employed to identify potential predictors of PD. A *P*-value threshold of <.05 was considered statistically significant for all tests.

## 3. Results

### 3.1. Baseline characteristics

At baseline, 269 (2.0%) participants were diagnosed with PD, whereas 13,380 did not. Table 1 shows the baseline characteristics of the 13,649 study participants. Participants in the Parkinson group were significantly older, with a mean age of 69.19 ± 9.91 years, compared to the non-Parkinson group, which had a mean age of 61.96 ± 9.18 years (*P* < .05). The PD’s group had a slightly higher proportion of males (56.1%) compared to the non-Parkinson group (49.3%; *P* = .032). The incidence of comorbidities was notably higher among participants with PD. Liver disease prevalence was significantly greater in Parkinson patients (13.0%) compared to controls (7.4%, *P* = .001). Lung disease was more prevalent in Parkinson patients (26.0% vs 13.7%, *P* < .05). Heart disease was also significantly higher in Parkinson cases (43.5%) versus non-cases (20.3%, *P* < .05). Similarly, the prevalence of stroke was markedly elevated in Parkinson patients (26.0%) compared to non-cases (5.7%, *P* < .05). Kidney disease (20.8% vs 10.9%), digestive disease (45.7% vs 31.3%), mental illness (16.0% vs 2.0%), memory impairment (100% vs 3.0%), arthritis (53.2% vs 36.8%), asthma (15.6% vs 5.4%), cancer (4.5% vs 2.4%), diabetes (26.0% vs 14.7%), hypertension (58.0% vs 38.0%), and dyslipidemia (43.9% vs 27.4%) all showed significantly higher frequencies in the Parkinson group (all *P* < .05).

**Table 1:** Baseline characteristics of the Parkinson and non-Parkinson groups.

Characteristics	Non-Parkinson (n = 13,380)	Parkinson (n = 269)	*P*-value
Gender			**.032**
Male	6603 (49.3)	151 (56.1)	
Female	6777 (50.7)	118 (43.9)	
Age	61.96 ± 9.18	69.19 ± 9.91	***P* < .05**
Liver disease			**.001**
Yes	991 (7.4)	35 (13.0)	
No	12,389 (92.6)	234 (87.0)	
Lung disease			***P* < .05**
Yes	1838 (13.7)	70 (26.0)	
No	11,542 (86.3)	199 (74.0)	
Heart disease			***P* < .05**
Yes	2722 (20.3)	117 (43.5)	
No	10,658 (79.7)	152 (56.5)	
Stroke			***P* < .05**
Yes	769 (5.7)	70 (26.0)	
No	12,611 (94.3)	199 (74.0)	
Kidney disease			***P* < .05**
Yes	1455 (10.9)	56 (20.8)	
No	11,925 (89.1)	213 (79.2)	
Digestive disease			***P* < .05**
Yes	4188 (31.3)	123 (45.7)	
No	9192 (68.7)	146 (54.3)	
Mental illness			***P* < .05**
Yes	266 (2.0)	43 (16.0)	
No	13,114 (98.0)	226 (84.0)	
Memory impairment			***P* < .05**
Yes	404 (3.0)	269 (100.0)	
No	12,976 (97.0)	0 (0.0)	
Arthritis			***P* < .05**
Yes	4929 (36.8)	143 (53.2)	
No	8451 (63.2)	126 (46.8)	
Asthma			***P* < .05**
Yes	726 (5.4)	42 (15.6)	
No	12,654 (94.6)	227 (84.4)	
Cancer			**.044**
Yes	317 (2.4)	12 (4.5)	
No	13,063 (97.6)	257 (95.5)	
Diabetes			***P* < .05**
Yes	1967 (14.7)	70 (26.0)	
No	11,413 (85.3)	199 (74.0)	
Hypertension			***P* < .05**
Yes	5088 (38.0)	156 (58.0)	
No	8292 (62.0)	113 (42.0)	
Dyslipidemia			***P* < .05**
Yes	3661 (27.4)	118 (43.9)	
No	9719 (72.6)	151 (56.1)	
Currently working			***P* < .05**
Yes	9180 (68.6)	107 (39.8)	
No	4200 (31.4)	162 (60.2)	
Currently retired			**.018**
Yes	2723 (20.4)	71 (26.4)	
No	10,657 (79.6)	198 (73.6)	
Living in rural area			.787
Yes	7574 (56.6)	155 (57.6)	
No	5806 (43.4)	114 (42.4)	
Vigorous physical activity			***P* < .05**
Yes	5002 (37.4)	61 (22.7)	
No	8378 (62.6)	208 (77.3)	
Moderate physical activity			***P* < .05**
Yes	8112 (60.6)	116 (43.1)	
No	5268 (39.4)	153 (56.9)	
Light physical activity			***P* < .05**
Yes	10,803 (80.7)	177 (65.8)	
No	2577 (19.3)	92 (34.2)	
Currently drinking alcohol			***P* < .05**
Yes	5303 (39.6)	67 (24.9)	
No	8077 (60.4)	202 (75.1)	
Ever smoked			.302
Yes	5919 (44.2)	110 (40.9)	
No	7461 (55.8)	159 (59.1)	
Currently smoking			**.001**
Yes	3671 (27.4)	49 (18.2)	
No	9709 (72.6)	220 (81.8)	
Visited clinic (last month)			***P* < .05**
Yes	2712 (20.3)	84 (31.2)	
No	10,668 (79.7)	185 (68.8)	
Hospitalized (last year)			***P* < .05**
Yes	2346 (17.5)	100 (37.2)	
No	11,034 (82.5)	169 (62.8)	
Has pension insurance			***P* < .05**
Yes	6944 (51.9)	184 (68.4)	
No	6436 (48.1)	85 (31.6)	
Has health insurance			.167
Yes	12,918 (96.5)	255 (94.8)	
No	462 (3.5)	14 (5.2)	
Urban employee insurance			.591
Yes	2198 (16.4)	48 (17.8)	
No	11,182 (83.6)	221 (82.2)	
Urban-rural resident insurance			1
Yes	1232 (9.2)	25 (9.3)	
No	12,148 (90.8)	244 (90.7)	
Urban resident insurance			.078
Yes	655 (4.9)	20 (7.4)	
No	12,725 (95.1)	249 (92.6)	
New rural cooperative insurance			**.027**
Yes	8514 (63.6)	153 (56.9)	
No	4866 (36.4)	116 (43.1)	
Public medical insurance			.49
Yes	161 (1.2)	5 (1.9)	
No	13,219 (98.8)	264 (98.1)	
Other medical insurance			.852
Yes	157 (1.2)	4 (1.5)	
No	13,223 (98.8)	265 (98.5)	
Monthly social participation			**.001**
Yes	6378 (47.7)	100 (37.2)	
No	7002 (52.3)	169 (62.8)	
Living with children			.945
Yes	8652 (64.7)	175 (65.1)	
No	4728 (35.3)	94 (34.9)	
Weekly face-to-face with children			***P* < .05**
Yes	3365 (25.1)	101 (37.5)	
No	10,015 (74.9)	168 (62.5)	
Weekly remote contact with children			***P* < .05**
Yes	7015 (52.4)	108 (40.1)	
No	6365 (47.6)	161 (59.9)	
Weekly any contact with children			.636
Yes	9258 (69.2)	182 (67.7)	
No	4122 (30.8)	87 (32.3)	
Received any aid from children			.326
Yes	10,902 (81.5)	226 (84.0)	
No	2478 (18.5)	43 (16.0)	
Gave any aid to children			.37
Yes	7998 (59.8)	153 (56.9)	
No	5382 (40.2)	116 (43.1)	
Polluting fuel for heating			.217
Yes	4178 (31.2)	94 (34.9)	
No	9202 (68.8)	175 (65.1)	
Polluting fuel for cooking			.202
Yes	3064 (22.9)	71 (26.4)	
No	10,316 (77.1)	198 (73.6)	
Has tap water			**.024**
Yes	12,277 (91.8)	236 (87.7)	
No	1103 (8.2)	33 (12.3)	
Has electricity			**.027**
Yes	13,300 (99.4)	264 (98.1)	
No	80 (0.6)	5 (1.9)	
Unhygienic toilet			.712
Yes	3944 (29.5)	76 (28.3)	
No	9436 (70.5)	193 (71.7)	
Poor building materials			.429
Yes	1043 (7.8)	25 (9.3)	
No	12,337 (92.2)	244 (90.7)	
Hukou (2-category)			.19
Yes	9566 (71.5)	182 (67.7)	
No	3814 (28.5)	87 (32.3)	
Any weekly physical activity			***P* < .05**
Yes	12,350 (92.3)	202 (75.1)	
No	1030 (7.7)	67 (24.9)	
Moderate or vigorous weekly activity			***P* < .05**
Yes	5002 (37.4)	61 (22.7)	
No	8378 (62.6)	208 (77.3)	

Bold value indicates statistical significance (*P* < .05).

The dataset was randomly divided into training (n = 9650) and validating (n = 3999) groups (Table 2). Baseline characteristics of the train and test groups. Table 2 shows the baseline characteristics of the train and test groups. No statistically significant differences were found between these groups for age (62.0 ± 9.24 years training vs 62.2 ± 9.45 years testing, *P* = .123), Parkinson prevalence (1.8% training vs 2.4% testing, *P* = .324), liver disease (7.4% training vs 7.9% testing, *P* = .288), cancer (2.3% training vs 2.6% testing, *P* = .454), mental illness (2.2% training vs 2.5% testing, *P* = .257), and memory-related disease (4.9% training vs 5.1% testing, *P* = .708). However, the prevalence of certain comorbidities showed significant differences. Lung disease prevalence was higher in the testing group (16.3% vs 13.0%, *P* < .05). Heart disease was slightly more common in the training set (21.3% vs 19.6%, *P* = .022), and stroke prevalence was greater in the training group (6.6% vs 5.1%, *P* = .002). Kidney disease prevalence was higher in the testing group (13.1% vs 10.2%, *P* < .05), as was digestive disease (35.3% vs 30.0%, *P* < .05) and arthritis (43.6% vs 34.5%, *P* < .05).

**Table 2:** Baseline characteristics of the train and test groups.

Characteristics	Train (n = 9650)	Test (n = 3999)	*P*-value
Age	61.99 ± 9.24	62.23 ± 9.45	.123
Parkinson disease			.324
Yes	173 (1.8)	96 (2.4)	
No	9477 (98.2)	3903 (97.6)	
Cancer			.454
Yes	226 (2.3)	103 (2.6)	
No	9424 (97.7)	3896 (97.4)	
Lung disease			***P* < .05**
Yes	1256 (13.0)	652 (16.3)	
No	8394 (87.0)	3347 (83.7)	
Liver disease			.288
Yes	710 (7.4)	316 (7.9)	
No	8940 (92.6)	3683 (92.1)	
Heart disease			**.022**
Yes	2057 (21.3)	782 (19.6)	
No	7593 (78.7)	3217 (80.4)	
Stroke			**.002**
Yes	634 (6.6)	205 (5.1)	
No	9016 (93.4)	3794 (94.9)	
Kidney disease			***P* < .05**
Yes	987 (10.2)	524 (13.1)	
No	8663 (89.8)	3475 (86.9)	
Digestive (stomach) disease			***P* < .05**
Yes	2898 (30.0)	1413 (35.3)	
No	6752 (70.0)	2586 (64.7)	
Mental illness			.257
Yes	209 (2.2)	100 (2.5)	
No	9441 (97.8)	3899 (97.5)	
Memory-related disease			.708
Yes	471 (4.9)	202 (5.1)	
No	9179 (95.1)	3797 (94.9)	
Arthritis			***P* < .05**
Yes	3327 (34.5)	1745 (43.6)	
No	6323 (65.5)	2254 (56.4)	
Asthma			.206
Yes	527 (5.5)	241 (6.0)	
No	9123 (94.5)	3758 (94.0)	
Social visit/interacting with friends			.339
Yes	3337 (34.6)	1348 (33.7)	
No	6313 (65.4)	2651 (66.3)	
Playing mahjong/board or card games/community activity room			.474
Yes	1665 (17.3)	669 (16.7)	
No	7985 (82.7)	3330 (83.3)	
Social visit/interacting with friends			.339
Yes	3337 (34.6)	1348 (33.7)	
No	6313 (65.4)	2651 (66.3)	
Playing mahjong/board or card games/community activity room			.474
Yes	1665 (17.3)	669 (16.7)	
No	7985 (82.7)	3330 (83.3)	
Unpaid help to non-household relatives/friends/neighbors			.232
Yes	1644 (17.0)	716 (17.9)	
No	8006 (83.0)	3283 (82.1)	
Dancing/exercising/Qigong in park or elsewhere			.334
Yes	815 (8.4)	317 (7.9)	
No	8835 (91.6)	3682 (92.1)	
Participating in organization activities			.999
Yes	283 (2.9)	118 (3.0)	
No	9367 (97.1)	3881 (97.0)	
Volunteer or charity activities			.674
Yes	371 (3.8)	147 (3.7)	
No	9279 (96.2)	3852 (96.3)	
Attending school or training courses			.851
Yes	189 (2.0)	81 (2.0)	
No	9461 (98.0)	3918 (98.0)	
Other social activities			.766
Yes	191 (2.0)	83 (2.1)	
No	9459 (98.0)	3916 (97.9)	
ADL: dressing			.506
Yes	554 (5.7)	242 (6.1)	
No	9096 (94.3)	3757 (93.9)	
ADL: bathing			.125
Yes	555 (5.8)	258 (6.5)	
No	9095 (94.2)	3741 (93.5)	
ADL: eating			.059
Yes	201 (2.1)	105 (2.6)	
No	9449 (97.9)	3894 (97.4)	
ADL: getting in/out of bed			**.007**
Yes	534 (5.5)	270 (6.8)	
No	9116 (94.5)	3729 (93.2)	
IADL: housekeeping			**.041**
Yes	1054 (10.9)	486 (12.2)	
No	8596 (89.1)	3513 (87.8)	
IADL: preparing meals			.36
Yes	684 (7.1)	302 (7.6)	
No	8966 (92.9)	3697 (92.4)	
IADL: shopping for groceries			.141
Yes	457 (4.7)	214 (5.4)	
No	9193 (95.3)	3785 (94.6)	
IADL: using telephone			.611
Yes	490 (5.1)	194 (4.9)	
No	9160 (94.9)	3805 (95.1)	
IADL: taking medication			.584
Yes	370 (3.8)	162 (4.1)	
No	9280 (96.2)	3837 (95.9)	
IADL: managing finances			.814
Yes	644 (6.7)	272 (6.8)	
No	9006 (93.3)	3727 (93.2)	
Social visit/interacting with friends			.339
Yes	3337 (34.6)	1348 (33.7)	
No	6313 (65.4)	2651 (66.3)	
Playing mahjong/board or card games/community activity room			.474
Yes	1665 (17.3)	669 (16.7)	
No	7985 (82.7)	3330 (83.3)	
Unpaid help to non-household relatives/friends/neighbors			.232
Yes	1644 (17.0)	716 (17.9)	
No	8006 (83.0)	3283 (82.1)	
Dancing/exercising/Qigong in park or elsewhere			.334
Yes	815 (8.4)	317 (7.9)	
No	8835 (91.6)	3682 (92.1)	
Participating in organization activities			.999
Yes	283 (2.9)	118 (3.0)	
No	9367 (97.1)	3881 (97.0)	
Volunteer or charity activities			.674
Yes	371 (3.8)	147 (3.7)	
No	9279 (96.2)	3852 (96.3)	
Attending school or training courses			.851
Yes	189 (2.0)	81 (2.0)	
No	9461 (98.0)	3918 (98.0)	
Other social activities			.766
Yes	191 (2.0)	83 (2.1)	
No	9459 (98.0)	3916 (97.9)	
ADL: dressing			.506
Yes	554 (5.7)	242 (6.1)	
No	9096 (94.3)	3757 (93.9)	
ADL: bathing			.125
Yes	555 (5.8)	258 (6.5)	
No	9095 (94.2)	3741 (93.5)	
ADL: eating			.059
Yes	201 (2.1)	105 (2.6)	
No	9449 (97.9)	3894 (97.4)	

ADL = activities of daily living.

Bold value indicates statistical significance (*P* < .05).

### 3.2. Feature selection outcomes

**Figure 2.**
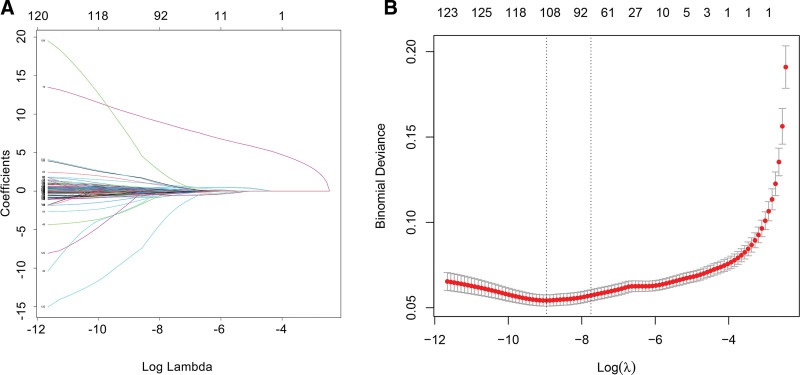
(A) LASSO coefficient paths for 120 candidate predictors. (B) 10-fold cross-validation curve showing mean-squared error (MSE). The left dashed vertical line indicates the λ value minimizing MSE (lambda.min), and the right dashed line indicates the most regularized model within 1 standard error of the minimum (lambda.1se). LASSO = least absolute shrinkage and selection operator.

The Boruta feature selection process identified multiple important predictors. Activities of daily living difficulties, specifically bathing, dressing, and eating, consistently demonstrated high importance scores. Cognitive function measures, including memory, orientation, and executive functions, were similarly identified as critical predictors by the Boruta algorithm. Figure 2 shown the LASSO regression method further refined the selection, highlighting cognitive function scores, liver disease, birth month, gender, and total metabolic equivalent (MET) from physical activity as significant predictors at the optimal lambda.min. The mRMR algorithm underscored the importance of minimizing redundancy while maintaining maximum relevance among these variables, resulting in a streamlined and highly predictive set of predictors.

### 3.3. Important predictive variables

**Figure 3.**
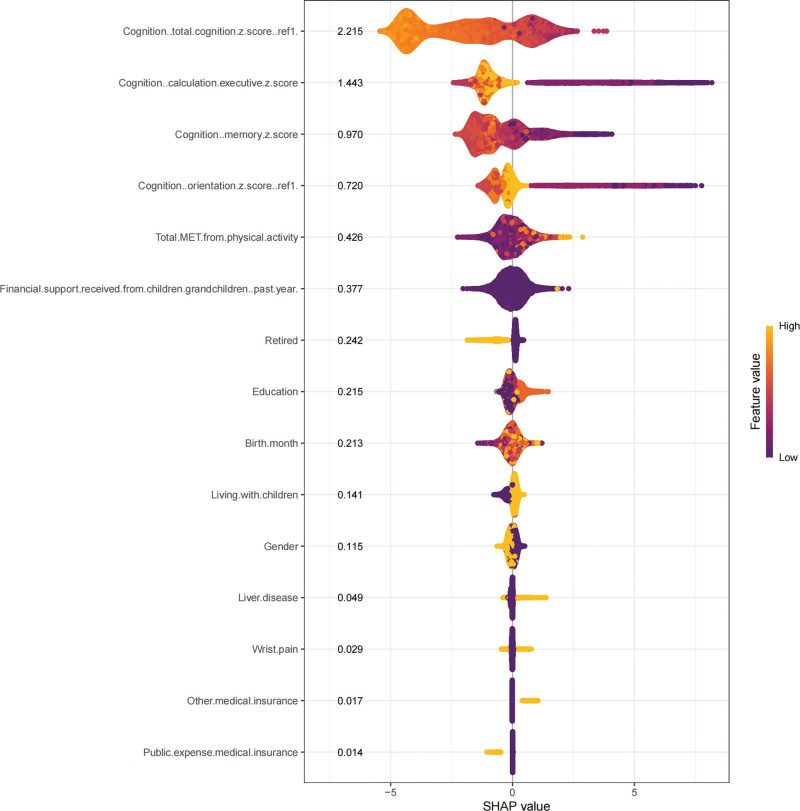
SHAP beeswarm plot for the XGBoost model, illustrating the distribution of SHAP values for each feature across all observations. Color intensity represents the feature value, highlighting its impact on PD risk prediction. PD = Parkinson disease, SHAP = SHapley Additive exPlanations, XGBoost = extreme gradient boosting.

Feature importance rankings were cross-validated through 1000 bootstrap iterations to ensure stability, with SHapley Additive exPlanations (SHAP) (Fig. 3) value distributions visualized through force plots and dependence diagrams. Key predictors identified from the XGBoost analysis included wrist pain, retirement status, financial support from family members, cognitive function scores (particularly memory, calculation, and executive functions), living arrangements, education level, and levels of physical activity. These variables were consistently ranked high in terms of predictive importance, underscoring their critical roles in identifying PD. The variable importance analysis from the XGBoost model highlighted the significance of socioeconomic factors such as financial support and retirement status, indicating a potential link between socioeconomic stability and PD progression or identification. Cognitive variables, including scores on memory and executive functions, reaffirmed the neurological implications of PD, emphasizing the need for comprehensive cognitive assessment in predictive modeling. Physical activity levels, measured by total MET scores, further reinforced the importance of lifestyle factors in PD prediction. Higher physical activity levels appeared inversely associated with PD likelihood, supporting existing evidence advocating physical activity as protective against neurological decline.

### 3.4. XGBoost model performance

**Figure 4.**
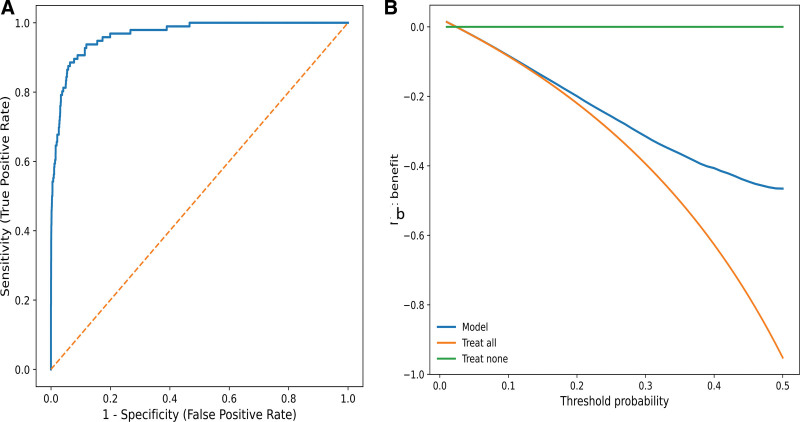
Discrimination and clinical utility of the mRMR-XGBoost prediction model in the validation set. (A) Receiver operating characteristic (ROC) curve illustrating model discrimination for Parkinson disease. The diagonal dashed line denotes chance-level discrimination. The model achieved an AUC of 0.967 (95% CI: 0.962–0.972). (B) Decision curve analysis (DCA) evaluating clinical utility across a range of threshold probabilities. The model (blue line) is compared with treat-all and treat-none strategies (orange and green lines), demonstrating the net benefit of using the model for decision-making over clinically relevant thresholds. AUC = area under the curve; CI = confidence interval; DCA = decision curve analysis; ROC = receiver operating characteristic, XGBoost = extreme gradient boosting.

The XGBoost-based prediction model demonstrated excellent performance in the validation set (Table 3). The model achieved a specificity of 94.7%, indicating a strong ability to correctly identify individuals without PD, and a sensitivity of 82.0%. The balanced accuracy was 0.884, providing an imbalance-robust summary of performance across both classes. The ROC analysis yielded an AUC of 0.967 (95% confidence interval: 0.962–0.972), supporting outstanding discrimination. In addition, the positive predictive value was 81.5%, the negative predictive value was 94.9%, and the F1-score was 81.7%, suggesting balanced performance across key classification metrics. The ROC curve (Fig. 4A) provides a visual summary of the sensitivity–specificity trade-off across thresholds. To assess potential clinical utility, decision curve analysis (Fig. 4B) indicated that the model provides a higher net benefit than the “treat-all” and “treat-none” strategies across a range of clinically relevant threshold probabilities.

**Table 3:** Performance metrics (specificity, sensitivity, balanced accuracy, AUC (95% CI), PPV, NPV, and F1-score) of the mRMR + XGBoost model in the validation set.

Validate set	mRMR + XGBoost
Specificity	0.947
Sensitivity	0.820
Balanced accuracy	0.883
AUC (95% CI)	0.967 (0.962–0.972)
PPV	0.815
NPV	0.949
F1-score	0.817

AUC = area under the curve, CI = confidence interval, NPV = negative predictive value, PPV = positive predictive value, XGBoost = extreme gradient boosting.

## 4. Discussion

Our study developed a robust predictive model for assessing the risk of PD among the middle-aged and elderly population in China by integrating multidimensional variables from the CHARLS cohort. The XGBost algorithm, optimized through ensemble feature selection (Boruta, LASSO, mRM), achieved exceptional discriminative performance (AUC = 0.967, 95% confidence interval: 0.962–0.972), outperforming existing PD prediction tools.^[19,20]^ For example, a previous PD prediction model using administrative claims data achieved an AUC of ~0.85,^[19]^ and a ML model based on nationwide health screenings reported AUC values around 0.74 to 0.78.^[20]^ These figures are substantially lower than the AUC of 0.967 achieved by our model. However, this exceptional performance should be interpreted with caution given the potential for overfitting. The relatively small number of PD cases and class imbalance in our dataset may limit the model’s robustness and generalizability to other populations, underscoring the need for external validation. Key indicators included cognitive impairments such as memory, orientation, and executive functions, alongside socioeconomic factors like financial assistance and retirement status, as well as existing health conditions, notably liver disease. Notably, modifiable lifestyle factors, including physical activity levels (total MET scores), emerged as beneficial predictors, consistent with the mechanistic understanding of exercise-induced neuroprotection.^[21,22]^ Furthermore, wrist pain, education level, and medical insurance were identified as novel yet clinically relevant variables, highlighting their potential utility as a screening tool in both clinical and public health contexts.

Our results not only align with but also significantly build upon previous studies across 3 key areas. Firstly, population-based studies indicate that cognitive changes may precede the diagnosis of PD, with cognitive dysfunction being a prodromal marker of PD.^[23]^ Furthermore, prospective studies have demonstrated cognitive decline in patients with prodromal or high-risk PD.^[24,25]^ Neuroimaging evidence shows that reduced basal forebrain volume correlates with cognitive deficits across various domains, including newly diagnosed and advanced-stage PD patients.^[26,27]^ The predictive importance of cognitive impairment further underscores these findings. Secondly, cohort studies have demonstrated a link between low physical activity and PD risk, with the highest level of physical activity associated with a 34% reduced risk compared to the lowest level.^[28]^ Thirdly, individuals with lower socioeconomic status typically face a higher risk of developing PD, indicating greater susceptibility to diverse adverse experiences throughout the life course.^[17]^

The identified predictors align with the pathophysiological mechanisms underlying PD. In humans, abnormalities in the noradrenergic^[29]^ and cholinergic^[30]^ pathways are associated with cognitive dysfunction in PD, even in the early stages of the disease.^[31]^ The cognitive decline observed in prodromal PD may be partially mediated by early pathological changes or non-dopaminergic systems.^[32]^ Animal studies support this notion; in a mouse model of PD, cognitive impairments, such as deficits in spatial learning and new object recognition, were observed prior to the onset of motor abnormalities and the loss of dopaminergic neurons.^[33]^ Research involving animals has demonstrated the protective benefits of physical exercise in mitigating the risk of developing PD. In studies using rodent models, imposed physical activity has been observed to increase striatal glial-derived neurotrophic factor levels, safeguarding dopamine neurons.^[34]^ Furthermore, this activity might diminish the proportion of dopamine transporters to cystic monoamine transporters, making dopamine neurons more resilient against toxic substances.^[35]^ Our research indicates that family financial support may play a protective role by alleviating psychological and social stress, thereby reducing the risk of PD. Chronic stress can activate the hypothalamic–pituitary–adrenal axis and promote neuroinflammation, thereby increasing the risk of PD.^[36]^ Consequently, socioeconomic factors and access to medical resources may modulate the risk of PD through psychosocial stress. The existing literature on the association between liver disease and PD is limited. However, it suggests that dysregulation of the gut–liver–brain axis, potentially due to altered bile acid metabolism, may contribute to an increased risk of developing PD.^[37]^ Using wrist pain as a predictive indicator represents a novel approach. Although musculoskeletal symptoms are prevalent in PD, the underlying mechanisms require further validation.

Furthermore, the integration of SHAP-based explainability in our model provides substantial clinical interpretability and practical value. The SHAP analysis allows us to explain individual predictions by showing how each feature contributes to a given patient’s risk, which can help clinicians trust the model’s output and act on the identified risk factors. For instance, if a patient is flagged as high-risk largely due to low physical activity or lack of social support, these findings could prompt targeted interventions (such as exercise programs or community support) to potentially mitigate the risk. By highlighting key contributors on a per-patient basis, the explainable AI component makes our tool more actionable in clinical screening workflows, as it not only identifies high-risk individuals but also suggests modifiable targets for early preventive strategies.

Although our research has advanced the risk stratification of PD, some limitations warrant consideration. Firstly, despite rigorous sampling methods, the limited number of PD cases (n = 269) restricts the feasibility of subgroup analyses, and it also increases the risk of model overfitting, potentially limiting its generalizability. Secondly, the diagnosis of PD in the CHARLS database is based on self-reported data, which may introduce potential misclassification bias. Thirdly, the model does not account for genetic factors, such as LRRK2 mutations, and biomarkers found in cerebrospinal fluid and blood, both of which could enhance prediction accuracy.

## 5. Conclusions

This study enhances the prediction of PD by integrating cognitive, motor, socioeconomic, and comorbidity factors within a ML framework. The model exhibits a high AUC and identifies clinically significant predictive factors, emphasizing its practicality for early detection and risk stratification. Future research should focus on validating the model across diverse populations, incorporating genetic and biomarker data, and clarifying the mechanistic links between socioeconomic stressors and PD pathogenesis. Interventions targeting modifiable risk factors, such as promoting physical activity and reducing economic disparities, may delay PD onset and improve patient outcomes.

## Acknowledgments

We extend our gratitude to the National Institute on Aging’s Behavioral and Social Research Division, the Natural Science Foundation of China, the World Bank, and Peking University for their financial support. We also express our appreciation to the CHARLS research and field teams, as well as all study participants, for their invaluable contributions.

## Author contributions

**Conceptualization:** Li Ke, Ying Li, Sili Jiang, Lei Zhao.

**Data curation:** Li Ke, Ying Li, Sili Jiang, Lei Zhao.

**Formal analysis:** Li Ke, Lei Zhao.

**Investigation:** Li Ke.

**Software:** Li Ke, Lei Zhao.

**Supervision:** Li Ke.

**Writing – original draft:** Li Ke, Ying Li.

**Writing – review & editing:** Li Ke, Lei Zhao.
